# Malignancy Risk Assessment in Patients with Thyroid Nodules Using Classification and Regression Trees

**DOI:** 10.1155/2013/983953

**Published:** 2013-09-11

**Authors:** Shokouh Taghipour Zahir, Fariba Binesh, Mehrdad Mirouliaei, Elias Khajeh, Sina Noshad

**Affiliations:** ^1^Department of Clinical Pathology, Sadoughi Hospital, Shahid Sadoughi University of Medical Sciences, Yazd, Iran; ^2^Department of Endocrinology and Metabolism, Sadoughi Hospital, Shahid Sadoughi University of Medical Sciences, Yazd, Iran; ^3^Endocrinology and Metabolism Research Center (EMRC), Valiasr Hospital, Tehran University of Medical Sciences, Tehran, Iran

## Abstract

*Purpose.* We sought to investigate the utility of classification and regression trees (CART) classifier to differentiate benign from malignant nodules in patients referred for thyroid surgery. *Methods.* Clinical and demographic data of 271 patients referred to the Sadoughi Hospital during 2006–2011 were collected. In a two-step approach, a CART classifier was employed to differentiate patients with a high versus low risk of thyroid malignancy. The first step served as the screening procedure and was tailored to produce as few false negatives as possible. The second step identified those with the lowest risk of malignancy, chosen from a high risk population. Sensitivity, specificity, positive and negative predictive values (PPV and NPV) of the optimal tree were calculated. *Results.* In the first step, age, sex, and nodule size contributed to the optimal tree. Ultrasonographic features were employed in the second step with hypoechogenicity and/or microcalcifications yielding the highest discriminatory ability. The combined tree produced a sensitivity and specificity of 80.0% (95% CI: 29.9–98.9) and 94.1% (95% CI: 78.9–99.0), respectively. NPV and PPV were 66.7% (41.1–85.6) and 97.0% (82.5–99.8), respectively. *Conclusion.* CART classifier reliably identifies patients with a low risk of malignancy who can avoid unnecessary surgery.

## 1. Introduction

Thyroid nodules are common findings in clinical practice. It is estimated that 4% to 7% of adults in the Unites States have palpable nodules upon examination [1]. With the advent of ultrasonographic (US) techniques for assessment of the thyroid gland, it is now believed that a nodule can be detected in as many as 67% of the normal population [2]. The first and foremost obstacle for the clinician is to determine whether the nodule is of a benign or malignant nature, albeit malignant nodules are quite rare, comprising approximately 5% of all nodules [1]. Fine needle aspiration (FNA) of the thyroid nodule has become the standard procedure for evaluation of nodule histopathology and is recommended as the main diagnostic strategy in several guidelines and published consensus agreements [3]. Nevertheless, FNA has multiple drawbacks inherent in the procedure itself [4], including the technique employed and the experience of the physician performing the aspiration [5, 6]. In an analysis of 4 703 FNAs performed in centers in New York and Texas, 10.4% of the FNAs were considered unsatisfactory, and another 17.4% were reported as either suspicious or indeterminate [7]. A considerable number of patients with equivocal findings are referred for surgery despite histopathological evaluation of the nodule after surgery revealing no malignancy [4]. It is estimated that around 75 000 surgeries for nodules with undetermined diagnoses are performed each year in the United States alone [8]; therefore, with a sound systematic approach, at least one third of these operations could be avoided [9]. 

Decision support systems that take advantage of computerized learning algorithms have been successfully applied in many areas of medicine and these have yielded diagnostic rates comparable or sometimes exceeding sophisticated diagnostic techniques and the clinical judgment of the physician [10–13]. Decision trees extracted from machine-based data mining techniques offer an unprecedented utility by translating large datasets into algorithms that can be directly applied in clinical settings [10]. In the present study, we sought to investigate the utility of classification and regression trees (CART) classifier in differentiating benign from malignant pathologies in a group of patients referred for evaluation. We hypothesized that a decision tree that has a high negative predictive value (NPV) could aid physicians to identify patients at low risk of thyroid carcinoma which could spare the patient from unnecessary surgery. 

## 2. Patients and Methods

### 2.1. Patients

Over a period of six years (2006–2011), all records and pathology slides of patients (*n* = 368) referred to the Sadoughi Hospital (Yazd, Iran) for surgery with a primary diagnosis of thyroid nodules were analyzed retrospectively. 

Patients with missing data on any clinical variable were excluded from the study. Evaluation of US variables of 68 patients was not possible (US examination was not performed before the operation or the US report was not available or was incomplete), and FNA results for 29 patients were inaccessible. Finally, the complete data of 271 subjects was collected and a final analysis was performed on this group. 

Clinical and demographic data, including patient's age, sex, clinical examination of the thyroid nodule, US reports, and laboratory evaluation of thyroid function test (TFT), were retrieved from the hospital archives. To create an unanimous presentation of US data, the following variables which were available in all reports were extracted: size of the nodule (if multiple nodules were detected, the size of the largest nodule), number of nodules (solitary, multiple), content (solid, cystic, or mixed), echogenicity (hyperechoic, isoechoic, or hypoechoic), microcalcifications (present or absent), and poorly defined margins (present or absent). 

### 2.2. Assessments

FNA biopsies were performed with the use of 25–27 gauge needles with either capillary action or aspiration techniques. Cytology samples were processed via a direct smear on a glass slide or by liquid-based preparations. For those thyroid nodules that were nonpalpable, predominantly cystic, or located posteriorly in the thyroid, a US guided FNA was performed. In patients with multiple thyroid nodules, only the largest and most solid-appearing nodule was selected for FNA biopsy, and the final diagnosis was based on histopathological examination of the surgical pathology of the selected nodule which had undergone FNA biopsy. Cytological diagnoses were reclassified into five categories: nondiagnostic, malignant, suspicious, indeterminate, and benign, according to the guidelines decided by the American Thyroid Association [14]. The specimen was considered to be sufficient if it contained a minimum of six groupings of well-preserved thyroid epithelial cells, consisting of at least 10 cells per group [15]. In cases with ambiguity in the reported diagnosis, the FNA slide was reviewed and corrections were made where necessary. 

Surgical specimens were reevaluated by a single experienced board-certified pathologist (Shokouh Taghipour), who was unaware of the clinical assessment, US results, or FNA findings of the patient. Histological diagnoses were classified into benign or malignant categories. Well-differentiated neoplasms without capsular invasion or definite nuclear changes were classified as benign, while minimally invasive follicular neoplasms were considered to be malignant. 

### 2.3. Statistical Analysis

Continuous variables are expressed as means ± standard deviations and categorical ones as numbers followed by percentages in parenthesis. Continuous variables between patients with benign and malignant nodules were compared using an independent *t*-test. To compare categorical variables between the two groups, a Pearson's chi square (or Fisher's exact test where necessary) was employed. Continuous variables, age and nodule size, were categorized into distinct groups according to optimal cut-off values provided by the decision tree. To further examine the probability of thyroid malignancy across the spectrum of age and size of the nodule, generalized additive models (GAM) were constructed [16]. Binomial distribution was assumed for the target variable (diagnosis: benign/malignant), with logit as the link function. The degree of freedom was automatically calculated according to the generalized cross-validation rule. 

To create a decision tree incorporating relevant clinical, laboratory, and US features of the thyroid nodule, CART which is a rule-based recursive partitioning method was employed [17]. Classification trees were derived using the Gini impurity measure as the splitting criterion. The sample was asymmetrically divided into approximately 75% and 25%. The larger sample, dubbed the learning sample, was used to extract the optimal decision tree. The sample containing 25% of the participants, dubbed the validation sample, served as the validation set. 

Based on theoretical background, a two-step approach for the identification of patients that can safely avoid surgery with a minimum risk of malignancy was established. The first step served as the screening procedure and was tailored in a manner to produce as few false negatives as possible. Therefore, allocation of the misclassification costs for false negatives was determined to be three times as high as false positives. This procedure directed the initial tree to exert an excellent sensitivity but yielded low specificity. Variables entered in this step were as follows: age, sex, nodule size, number of nodules, presence of goiter, TFT, and presence of alarm signs, including hoarseness, weight loss, family history of thyroid cancer, and lymphadenopathy. The optimal tree was selected on the basis of a trade-off between parsimony and cross-validation costs. Therefore, in the first step, the optimal tree was the most clinically sound parsimonious tree with cross-validation costs within 0.1 standard error of the tree automatically selected by the system. If such a tree was not available within the system-derived nested set of trees, the optimal tree was extracted using pruning of the system-generated optimal tree. The first step was designed to detect the population at highest risk of malignancy. However, the second step was directed in the opposite manner in order to identify those with the lowest risk of malignancy, chosen from a high risk population. These subjects are often the ones that undergo unnecessary surgery in clinical practice, with no malignancy detected on histopathological examination despite equivocal or suspicious findings on the FNA. 

In the second step, US derived variables were entered given their high specificity. These variables included nodule content (cystic, solid, mixed), echogenicity (hypoechoic, isoechoic, hyperechoic), poorly defined margins (present, absent), microcalcification (present, absent), and combined diads or triads of these variables using logical terms and/or. The process of selecting the optimal tree was identical to the previous step. The combined tree was then validated on the test set. In addition, it was also validated on a subset of test samples which had FNA results labeled nondiagnostic, indeterminate, or suspicious, to see if the derived algorithm would be useful in discriminating benign from malignant in cases where FNA findings are not conclusive. For the learning set, test set, and unsatisfactory FNA set, the sensitivity, specificity, positive predictive value (PPV), and NPV, along with their 95% confidence intervals (95% CI), were calculated. Univariate statistics were performed using SPSS version 18 (IBM corp., NY, USA). For GAM and CART, Statistica version 8 (StatSoft Inc., OK, USA) was used. 

## 3. Results

### 3.1. Univariate Statistics

A total of 271 subjects met the inclusion criteria. Patients' age ranged from 23 to 78 years (mean: 45.4 ± 13.9), and women comprised 81.5% of the subjects. Baseline characteristics of the study participants according to their thyroid nodule pathology are summarized in Table 1. Mean age of the subjects with benign and malignant pathologies did not differ significantly (44.6 ± 12.3 in the benign, and 46.9 ± 17.0 in the malignant group, *P* = 0.218). However, after classification of age into categories it was revealed that subjects in the malignant group are more likely to be younger than 30 or older than 60 years when compared with individuals in the benign group (*P* = 0.008). The female to male ratio was 4.5 : 1; men were more likely to present with malignant nodules (*P* = 0.011). On the other hand, subjects in the two groups did not differ significantly with respect to the presence of thyroid enlargement on examination, number of thyroid nodules, and TFT (Table 1). The sizes of the thyroid nodules were significantly different between the two groups; nodules of <2 cm were more likely to be malignant, whereas those of >5 cm were usually of benign pathology. 

**Figure 1:**
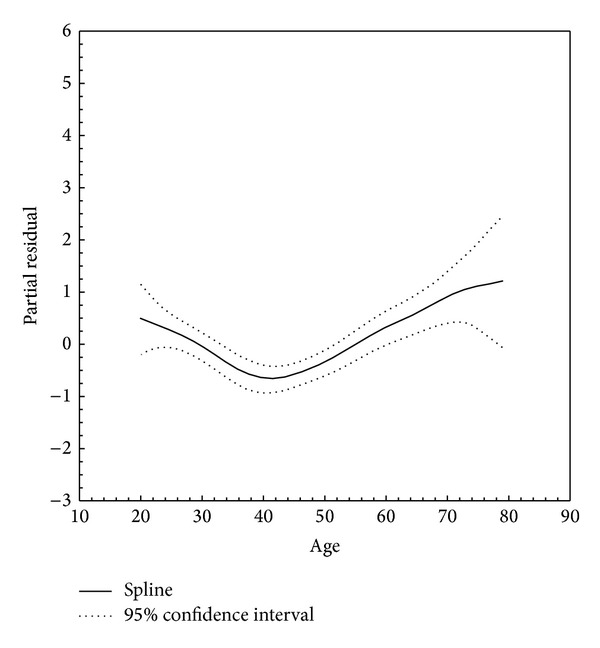
Smoothed association of age with thyroid cancer (non-linear *P* = 0.004).

**Figure 2:**
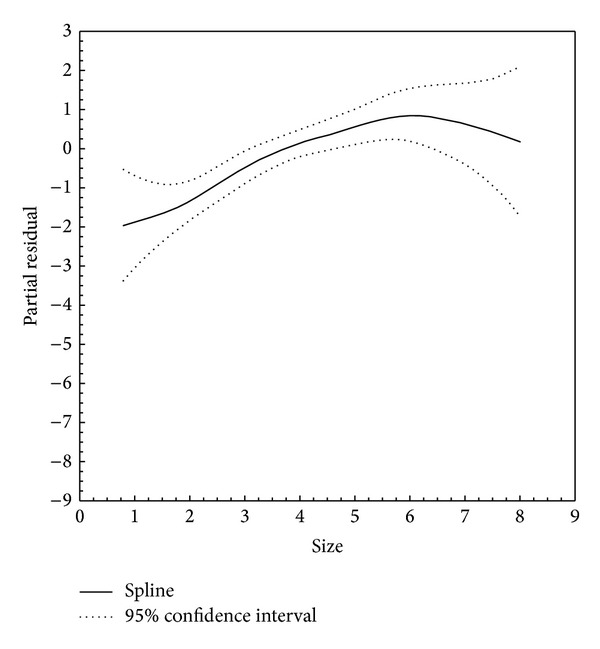
Smoothed association of nodule size with thyroid cancer (non-linear *P* = 0.220).

GAM showed that the association between age and thyroid cancer is better fitted using a curvilinear vector (nonlinear *P* = 0.004). Based on the drawn curve, a medium to high risk for malignancy is expected in the early 20s with a steady decline towards older age. After reaching the lowest point at around 40 years, the risk of malignancy then increases steadily, finally slowing after 70 years (Figure 1). Drawn spline of the nodule size also showed a complex curvilinear pattern, although this did not reach statistical significance (non-linear *P* = 0.220, Figure 2). 

The FNA categorized thyroid nodules into nondiagnostic (23 subjects, 8.5%), benign (52 subjects, 19.2%), malignant (45 subjects, 16.6%), suspicious (58 subjects, 21.4%), and indeterminate (93 subjects, 34.3%). Of note, four cases (8.9%) that were initially diagnosed by FNA as malignant turned out to be false positives. Moreover, eight cases (15.3%) that were classified as benign in FNA were in fact false negatives that turned out to be malignant carcinomas (seven papillary cell carcinomas, one follicular carcinoma). The majority of indeterminate FNA results proved to be multinodular goiter (49 subjects, 52.7%), follicular adenoma (39 subjects, 41.9%), and lymphocytic thyroiditis (4 subjects, 4.3%). 

The results of the histopathological diagnoses obtained following surgery are summarized in Table 2. In the majority of cases (195 subjects, 72%), patients had diagnoses compatible with benign pathology. The most frequent diagnostic category reported was multinodular goiter (133 subjects, 68.3%), followed by follicular adenoma (40 subjects, 20.5%), lymphocytic thyroiditis (nine subjects, 4.6%), and Hashimoto's thyroiditis (eight subjects, 4.1%). Furthermore, the most frequent type of malignancy comprising more than three quarters of all identified malignancies was papillary cell carcinoma. Other types of malignancy, in order of frequency, were follicular cell carcinoma, medullary cell carcinoma, Hurthle cell carcinoma, and carcinoma of anaplastic nature (Table 2). 

### 3.2. Decision Tree

**Figure 3:**
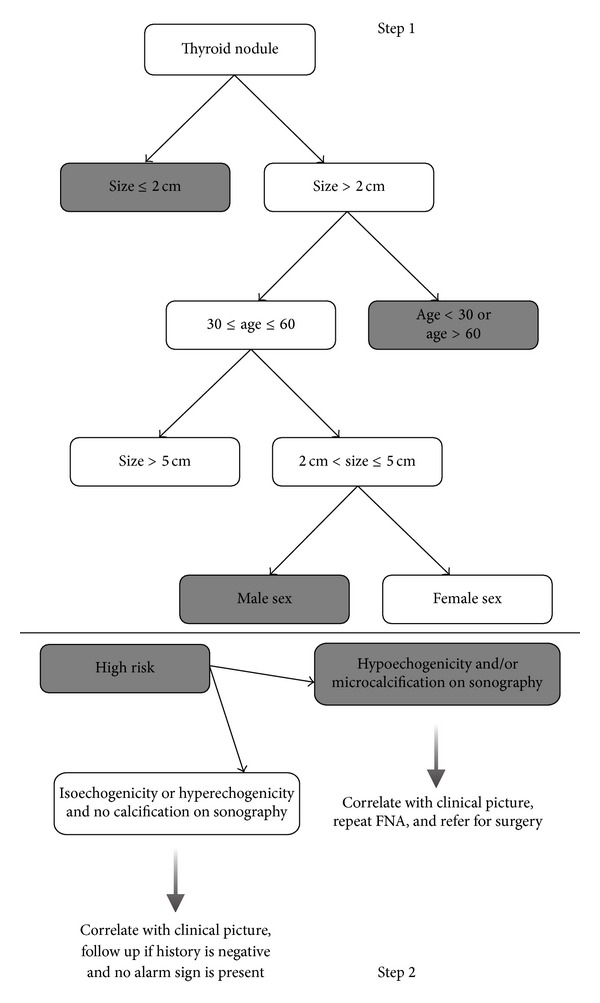
Optimal decision tree for stratifying patients based on their risk for thyroid cancer. Subjects categorized in the gray boxes (high-risk patients) in the first step are reevaluated in the second step using US findings. *Syntax*: if nodule size ≤2 cm *and* hypoechogenicity/microcalcification → high risk. If size >2 cm *and* (age <30 *or* >60) *and* hypoechogenicity/microcalcification → high risk. If (2≤ size ≤5) *and* (30≤ age ≤60) *and* male sex *and* hypoechogenicity/microcalcification → high risk. Otherwise → low risk.

Sensitivity, specificity, PPV and NPV of the optimal trees in steps 1 and 2, and combined in learning and test samples, and “inconclusive FNA” datasets are presented in Table 3. Of all the entered variables in the first step, only three, namely age, sex, and nodule size, contributed to construction of the optimal tree, with age having the highest discriminatory ability, followed by nodule size and sex. The derived optimal tree using the CART procedure is illustrated in Figure 3. 

All US variables were able to significantly discriminate between low versus high risk patients in a univariate analysis (solid content *P* = 0.033, hypoechogenicity *P* < 0.001, presence of poorly defined margins *P* < 0.001, and presence of microcalcification *P* = 0.003). Of the multiple combination of US variables examined in the second step, hypoechogenicity and/or calcification yielded the highest discriminatory ability, correctly identifying 43 out of 47 benign cases and 42 out of 51 malignant ones (Table 3 and Figure 3). In the final step, the two trees were combined to derive a final unified tree that yielded a sensitivity and specificity of 77.8 (95% CI: 64.1–87.5) and 97.1 (95% CI: 92.2–99.0), respectively. 

### 3.3. Validation on Test Sample and Subjects with Inconclusive FNA

The optimal tree derived from the learning sample was validated on the remaining 25% of the population (i.e., test sample). Although all indices of accuracy were lower in the test sample, the optimal tree retained a good NPV of 88.5% (95% CI: 77.2–94.5). The tree was further validated in a subset of test samples with inconclusive FNA results (*n* = 39). The tree correctly identified four out of five subjects with malignancy (PPV = 66.7, 95% CI: 41.1–85.6) and was able to accurately discriminate 32 out of the 34 benign cases examined (NPV = 97.0, 95% CI: 82.5–99.8). Based on these findings, a patient declared at low risk with the optimal tree has a 3% chance of malignancy. The false negative subject was a 68-year-old female, with a 3 cm nodule and no US features suggestive of malignancy, which turned out to be a follicular cell carcinoma. One of the two false positives was a 29-year-old woman with a 4.5 cm nodule and isoechogenicity on the sonography. The other was a 42-year-old male that had fine calcifications on US examination. 

## 4. Discussion

The rate of diagnosis of thyroid cancer has doubled in the past 30 years, despite no tangible decreases in mortality rates. These observations suggest that the increased incidence is more likely a result of overdiagnosis of early forms of thyroid malignancies rather than a true rise in its secular trends [18]. It is conceivable that overdiagnosis of thyroid malignancies translates into a sizable number of unnecessary thyroidectomies and complications associated with this type of surgery, along with detrimental effects on patients' quality of life [19]. 

FNA biopsy has become the procedure of choice, due to its accuracy, safety, and cost-effectiveness, for the initial evaluation of thyroid nodules and determination of malignancy risk [20]. The use of FNA biopsy has resulted in a 25% decrease in thyroidectomies of benign nodules [21]. Although FNA biopsy can be conveniently performed, obtaining adequate tissue, proper processing of the specimen, and accurate cytopathological interpretation of the prepared slides is highly dependent on the expertise and experience of the laboratory personnel and the pathologist in charge [22, 23]. It is estimated that up to 20% of obtained FNA samples yield nondiagnostic or insufficient results [24]. However, in the present study, a proportionately high rate of inadequate or nondiagnostic FNAs was identified, further confirming the notion that with low precision in diagnostic techniques, a high rate of unnecessary operations ensues. In the absence of an adequately obtained and interpreted FNA biopsy, special emphasis should be placed on history, physical examination, and US evaluation, with the aim of reaching a sound clinical decision for thyroid nodule management, thereby avoiding unnecessary surgery. Machine-based decision support systems with the ability to stratify patients based on malignancy risk are indispensable in this regard. In practical terms, a decision tree capable of providing a high NPV could reliably select a subgroup of patients with a low risk of malignancy. It should be noted, however, that previous experience has gathered evidence that physicians are often reluctant to incorporate such decision support systems into their daily clinical practice owing to the often perplexing and time-consuming nature of such strategies. Hence, it is imperative to develop an algorithm that while it is able to select pertinent variables essential for making a clinical diagnosis, it is also parsimonious at its core. It is only then that a derived decision tree is expected to be integrated into routine clinical practice. 

Taking these assumptions into consideration, in the present study we aimed to derive an optimal decision tree that is applicable to available clinical data using the CART classifier. Here, we observed that a final optimal tree with a sensitivity of approximately 80% and specificity of 94.0% can divide patients into two broad categories of high risk (66.7% risk for thyroid malignancy) and low risk (only 3% risk for cancer). Patients categorized as low risk by the optimal tree can safely be spared from immediate referral for surgery, provided there is no other suspicious findings revealed during the course of patient evaluation. Ippolito et al. [25] used neural network analysis to integrate cytological and clinical data in a multivariate classifier that is helpful in the diagnosis of thyroid nodules labeled indeterminate at FNA. The final model categorized patients into high risk (33% chance of malignancy) versus low risk (3% chance of malignancy). Of note, only cytological parameters contributed to the final model [25]. In another study, Stojadinovic et al. [26] created a clinical decision model employing Bayes' theorem. The model included nodule size, FNA cytology, US, and electrical impedance scanning of the thyroid, and this yielded positive and negative predictive values of 83% and 79%, respectively. Using the same method, Liu et al. [27] built a Bayesian network to combine US and demographic features capable of calculating malignancy risk in thyroid nodules. It was shown that the Bayesian analysis is as sensitive as two expert radiologists, who independently reviewed the US images and evaluated each case on a 5-point scale of suspicion for malignancy [27]. In comparison to the studies outlined above, our derived model provides acceptable NPV (97%) and PPV (66.7%).

CART is a machine-learning method which uses a binomial splitting criterion to classify data into decision trees. The splitting criterion of the CART uses a set of Yes/No questions to find the best point to split a sample of learning data into two distinct parts that have maximum homogeneity. The binomial partitioning which can be applied to both continuous and categorical variables is done recursively until all members of the learning sample are placed in the so-called leaves of the tree. 

Developed by Breiman, Friedman, Olshen, and Stone in 1984, CART has multiple advantages that set it apart from other competing algorithms [17]. From a statistical perspective, CART does not require the assumption of normality, easily handles outliers, and is computationally fast. From a clinical standpoint, CART provides algorithms that closely parallel the process of clinical reasoning. Furthermore, the derived algorithms are flexible and can be easily pruned to adjust to the setting they are being applied to. A major challenge, however, is to find balance between accuracy and parsimony of the developed decision algorithm. At times, the derived tree could be quite complex, consisting of tens of branches and leaves; any significant pruning would require forfeiting accuracy at the expense of parsimony and a balanced approach is not always feasible. Despite these limitations, in the past few years, CART has found its way into clinical research and has successfully been applied to enhance the accuracy of diagnosis of malignant tumors of the prostate, lung, and carcinomas of unknown primary [28–30]. 

Our final model showed that nodule size of equal to or less than 2 cm, patient's age of less than 30 years or more than 60 years, male sex, hypoechogenicity, and/or microcalcification documented by US infer susceptibility to thyroid cancer. For continuous variables (i.e., age and nodule size), GAM splines were drawn to investigate whether curvilinear patterns estimate a correlation with malignancy better than linear statistics. Indeed, GAM produced better capture of the observed patterns across the age spectrum (non-linear *P* = 0.004, Figure 1). Although not statistically significant, a GAM spline for nodule size also showed a complex curvilinear pattern (non-linear *P* = 0.220, Figure 2). These observations are in agreement with previous evidence demonstrating a higher risk of developing cancer in male gender, larger nodules, and extremes of age (<30 and 60> years) [31]. Furthermore, multiple US features have been nominated as potential predictors of thyroid carcinoma [32]. Previous studies have shown that hypoechogenicity and microcalcifications are present in 26%–87% and 26%–59% of all thyroid cancers, respectively [33]. Our findings also support the notion that US features are highly specific for differentiating between benign and malignant thyroid nodules [34–37]. In a multicenter study of 831 patients with thyroid nodules [34], marked hypoechogenicity and microcalcification demonstrated excellent specificity (92.2% and 90.8%, resp.), although it revealed low sensitivity (41.4% and 44.2%, resp.) for diagnosis of malignant thyroid nodules.

Few limitations in our study deserve to be discussed. First, only patients referred for surgical interventions were included in our study, and this selection bias may have resulted in overpresentation of patients in whom thyroid nodules turned out to be of a malignant nature. However, it should be noted that our derived model was specifically tailored to identify low risk patients who would not benefit from surgery, despite being nominated for other reasons. Therefore, the optimal tree developed in this study should be applied to clinical settings similar to ours. Indeed, physicians in their daily clinical practice usually find the question of “whether to refer suspicious patients to surgery or not” troublesome. Second, the retrospective nature of our study might have resulted in preferential inclusion of patients who have complete clinical, US, and histopathological assessments. It should also be noted that while our decision system yielded promising results when applied to the subset of patients with inconclusive FNA findings, since it was built on the general population of patients referred for thyroid nodule evaluation, it should only be applied in settings with similar clinical pictures and patient profiles.

Collectively, our study provides further evidence that machine-based decision support systems can accurately distinguish benign from malignant thyroid nodules. Optimal trees derived from a CART classifier are capable of using available clinical and US data in a multivariate manner and could help physicians reach a clinical decision in cases where ambiguity exists. Promising preliminary data obtained here needs to be externally validated on independent large datasets.

## Figures and Tables

**Table 1:** Descriptive characteristics of 271 patients referred for thyroid nodule evaluation between 2006 and 2011.

	Benign	Malignant	*P* value	Total
Age (years)			0.008	
<30	37 (19.0)	20 (26.3)		57 (21.0)
≥30 and ≤60	135 (69.2)	38 (50.0)		173 (63.8)
>60	23 (11.8)	18 (23.7)		41 (15.2)
Sex (women/men)	167/28	55/21	0.011	222/49
Thyroid enlargement, *n* (%)	110 (56.4)	46 (60.5)	0.538	156 (57.6)
Number of nodules, *n* (%)			0.191	
Single	182 (93.3)	74 (97.4)		256 (94.5)
Multiple	13 (6.7)	2 (2.6)		15 (5.5)
Nodule size, *n* (%)			<0.001	
<2 cm	4 (2.1)	10 (13.2)		15 (5.5)
2–5 cm	128 (65.6)	52 (68.4)		179 (66.0)
>5 cm	63 (32.3)	14 (18.4)		77 (28.5)
TFT, *n* (%)			0.288	
Hyperthyroid	9 (4.6)	2 (2.6)		11 (4.1)
Euthyroid	139 (71.3)	49 (64.5)		188 (69.4)
Hypothyroid	47 (24.1)	25 (32.9)		72 (26.5)
FNA, results *n* (%)			<0.001	
Nondiagnostic	16 (8.2)	7 (9.2)		23 (8.5)
Benign	44 (22.5)	8 (10.5)		52 (19.2)
Indeterminate	85 (43.6)	8 (10.5)		93 (34.3)
Suspicious	46 (23.6)	12 (15.8)		58 (21.4)
Malignant	4 (2.1)	41 (54.0)		45 (16.6)

Abbreviations: TFT: thyroid function test; FNA: fine needle aspiration.

**Table 2:** Pathologic diagnoses of thyroid nodules evaluated between 2006 and 2011.

Benign *n* (%)	
Multinodular goiter	133 (68.3)
Follicular adenoma	40 (20.5)
Lymphocytic thyroiditis	9 (4.6)
Hashimoto's thyroiditis	8 (4.1)
Hurthle cell adenoma	3 (1.5)
Graves' disease	2 (1.0)
**Total**	**195 (100)**

Malignant *n* (%)	
Papillary cell carcinoma	59 (77.7)
Follicular cell carcinoma	8 (10.5)
Medullary cell carcinoma	5 (6.6)
Hurthle cell carcinoma	2 (2.6)
Anaplastic Carcinoma	2 (2.6)
**Total**	**76 (100)**

**Table 3:** Sensitivity, specificity, and positive and negative predictive values of the optimal tree in discriminating benign from malignant pathologies.

	Learning sample (*n* = 190)	Test sample (*n* = 81)	Test sample with nondiagnostic, indeterminate, or suspicious FNA (*n* = 39)
First step			
Sensitivity	94.4 (84.9–98.1)	91.3 (70.5–98.5)	100.0 (46.3–1.0)
Specificity	65.4 (57.1–72.9)	56.9 (43.3–69.6)	61.8 (43.6–77.3)
PPV	52.1 (41.8–62.1)	45.7 (31.2–60.8)	27.8 (10.7–53.6)
NPV	96.7 (90.1–99.1)	94.3 (79.5–99.0)	100.0 (80.8–1.0)
Second step			
Sensitivity	82.3 (68.6–91.1)	76.2 (52.4–90.9)	80.0 (29.9–98.9)
Specificity	91.5 (78.7–97.2)	84.0 (63.1–94.7)	84.6 (53.7–97.3)
PPV	91.3 (78.3–97.2)	80.0 (55.7–93.4)	66.7 (41.1–85.6)
NPV	82.7 (69.2–91.3)	80.8 (60.0–92.7)	91.7 (59.7–99.6)
Decision tree			
Sensitivity	77.8 (64.1–87.5)	69.6 (47.0–85.9)	80.0 (29.9–98.9)
Specificity	97.1 (92.2–99.0)	93.1 (82.4–97.8)	94.1 (78.9–99.0)
PPV	91.3 (78.3–97.2)	80.0 (55.7–93.4)	66.7 (41.1–85.6)
NPV	91.7 (85.6–95.4)	88.5 (77.2–94.5)	97.0 (82.5–99.8)

Abbreviations: FNA: fine needle aspiration; PPV: positive predictive value; NPV: negative predictive value.
